# Individually addressable and spectrally programmable artificial atoms in silicon photonics

**DOI:** 10.1038/s41467-023-37655-x

**Published:** 2023-04-25

**Authors:** Mihika Prabhu, Carlos Errando-Herranz, Lorenzo De Santis, Ian Christen, Changchen Chen, Connor Gerlach, Dirk Englund

**Affiliations:** 1 Massachusetts Institute of Technology, Cambridge, MA USA; 2 University of Münster, Münster, Germany; 3 QuTech, Delft University of Technology, Delft, The Netherlands

**Keywords:** Quantum optics, Single photons and quantum effects, Silicon photonics

## Abstract

A central goal for quantum technologies is to develop platforms for precise and scalable control of individually addressable artificial atoms with efficient optical interfaces. Color centers in silicon, such as the recently-isolated carbon-related G-center, exhibit emission directly into the telecommunications O-band and can leverage the maturity of silicon-on-insulator photonics. We demonstrate the generation, individual addressing, and spectral trimming of G-center artificial atoms in a silicon-on-insulator photonic integrated circuit platform. Focusing on the neutral charge state emission at 1278 nm, we observe waveguide-coupled single photon emission with narrow inhomogeneous distribution with standard deviation of 1.1 nm, excited state lifetime of 8.3 ± 0.7 ns, and no degradation after over a month of operation. In addition, we introduce a technique for optical trimming of spectral transitions up to 300 pm (55 GHz) and local deactivation of single artificial atoms. This non-volatile spectral programming enables alignment of quantum emitters into 25 GHz telecommunication grid channels. Our demonstration opens the path to quantum information processing based on implantable artificial atoms in very large scale integrated photonics.

## Abstract (web summary)

Realising integrated photonic circuits containing isolated telecommunications-wavelength artificial atom single photon emitters is an outstanding challenge in quantum technologies. Here, the authors demonstrate how to embed optically tunable G-centers in silicon-on-insulator integrated circuits.

## Introduction

Artificial atoms in the solid state are leading candidates for spin-based quantum information processing due to their long spin coherence times and high-quality spin-photon interfaces^1–3^. However, traditional platforms based on diamond and silicon carbide face two critical challenges for large-scale quantum information processing: lack of monolithic manufacturability and inefficient optical interfacing with the optical fiber telecommunication bands. Efforts towards alleviating these limitations include hybrid integration^2,4,5^ and quantum frequency conversion^6^, although at the cost of greater optical loss (>7 dB at present^6^). Recently, a number of color centers in silicon^7–10^, such as the carbon-based G-center^8,10^, have emerged as promising qubit candidates, as they can be integrated natively with existing commercial silicon platforms and their telecom wavelength emission obviates the need for frequency conversion. Furthermore, the lack of a nuclear spin bath in isotopically purified ^28^Si has allowed demonstrations of electron and nuclear spin coherence *T*_2_ for donors in silicon exceeding 2 seconds^11^ and 39 minutes^12^, respectively.

**Fig. 1:**
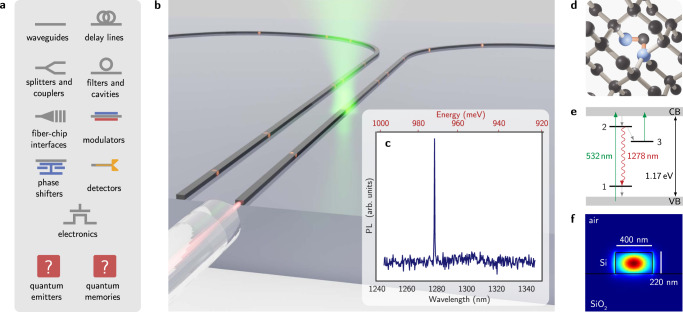
G-centers in silicon photonics as a scalable quantum photonic platform. **a** The silicon platform features technologically mature opto-electronics, but lacks integrated quantum emitters and memories. **b** Schematic showing our system, which integrates artificial atoms (G-centers) in silicon photonic waveguides. **c** Measured G-center photoluminescence (PL) spectrum through our waveguide, showing light emission in the O-band. **d** The G-center atomic structure consists of two substitutional carbon atoms (blue) bonded to an interstitial silicon and **e** has two optically addressable energy levels and a third metastable level within the silicon bandgap. **f** A finite-difference eigenmode simulation showing the electric field norm for our single-mode waveguide geometry.

An artificial atom platform based on the silicon G-center could immediately access a vast manufacturing and science toolkit, including the world’s most advanced complementary metal-oxide-semiconductor platforms, which already include carbon defects^13^ and large-scale patterning with nanometer-scale resolution^14^. Moreover, very large scale integrated silicon photonics can integrate millions of devices onto a single wafer, and already includes nearly all the necessary components for a full-stack quantum photonic system: low-loss passive components such as waveguides, splitters, fiber-to-chip interfaces^15^, and ultra-high quality factor cavities^16^; high-speed and cryogenically-compatible active modulators^17^ and phase shifters^18,19^; superconducting single-photon detectors^20^, and control electronics^21^ (Fig. 1a).

Although dense ensembles of silicon artificial atoms have been recently integrated into photonic waveguides^22,23^, the isolation of single silicon artificial atoms in photonic integrated circuits (PIC) remains a central challenge. While the integration of quantum emitters in PICs in other material systems has already resulted in a plethora of new physical breakthroughs and applications^24^, in silicon, a platform that combines individually-addressable G-centers with mature integrated photonics could open the door to industrial-scale quantum photonics. Such a technology has the potential to address the scalability challenges of quantum information processing.

Here we report monolithic integration of single artificial atoms—G-centers—in silicon PICs (Fig. 1b) and their optically-induced non-volatile spectral programming. Low temperature spectroscopy reveals single photon emission in the telecommunications O-band (Fig. 1c) with a narrow inhomogeneous distribution of 1.1 nm and spectral shifts up to 300 pm (55 GHz).

## Results

The silicon G-center consists of a pair of substitutional carbon atoms bonded to a silicon self-interstitial (C_s_-Si_i_-C_s_) within the silicon crystal lattice (see Fig. 1d). It exhibits a zero phonon line (ZPL) transition at 970 meV (1278 nm), resulting from an electron transition between *sp*-like orbitals localized at the Si_i_ atom (Fig. 1e) and features a spin triplet metastable state^25^. While the primary mechanism for above-band population of the excited state in G-centers is likely to be Shockley–Read–Hall recombination, additional non-radiative mechanisms such as surface recombination at electronically active surface states and Auger recombination are also known to affect carrier dynamics in silicon^26^ (a more detailed discussion can be found in Supplementary Note 8).

**Fig. 2:**
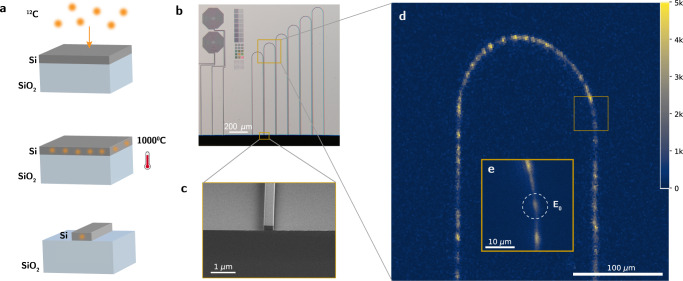
Fabrication and imaging of G-centers in silicon photonics. **a** Fabrication process for G-centers in silicon photonic waveguides. **b** Microscope image of our sample, showing several silicon photonic structures. **c** Scanning electron micrograph (SEM) of a cleaved facet of one of our waveguides. **d** A PL map taken under 10 μW 532 nm green continuous-wave excitation shows discrete light emitters coupled into the waveguide. **e** Emitter 0 under study.

The device under study consists of a silicon-on-insulator (SOI) waveguide designed for single-mode operation at 1278 nm, the ZPL of the G-center. Simulation results for the electric field distribution within the waveguide are shown in Fig. 1f. We fabricated our samples by a combination of commercial carbon implantation, thermal annealing, and foundry electron beam lithography and etching (see Fig. 2a and Methods). Our sample contains several waveguides and photonic structures. The waveguides in our sample (see Fig. 2b) start and end on a cleaved facet (Fig. 2c) and loop in a 63.5 μm radius bend.

We characterized the samples using low-temperature spectroscopy in a cryostat at a base temperature of 6 K. Using above band-gap laser excitation (wavelength *λ*_exc_ = 532 nm, NA = 0.55, and power *P*_in_ measured before the objective), we induced artificial atoms to emit into the waveguide. The waveguide-coupled optical emission from the emitters was subsequently collected with an edge-coupled lensed fiber at the cleaved chip facet. We band-pass filtered the fiber-collected emission between 1250 nm and 1550 nm to isolate the zero-phonon line (ZPL) and phonon sideband of the G-centers while attenuating residual pump light and other background. The collected emission was then detected with either a cryogenic superconducting-nanowire single-photon detector (SNSPD) system or an infrared spectrometer.

### Photoluminescence raster scans

We spatially locate our artificial atoms within the waveguides using photoluminescence (PL) raster scans. We scanned the focused continuous-wave green excitation along the chip plane and detected the filtered emission through the fiber-coupled waveguide with an electronically-gated SNSPD. Figure 2d shows PL signal in spatially-isolated loci along the waveguide, corresponding to single emitters or small ensembles of emitters. A range in brightness of the PL hotspots can be attributed to the presence of clusters of emitters in close proximity, which is confirmed through the observation of multiple distinct ZPL peaks in the PL spectra taken from these points (Supplementary Note 5 and Supplementary Fig. 5b).

### Spectral characterization of emitters

**Fig. 3:**
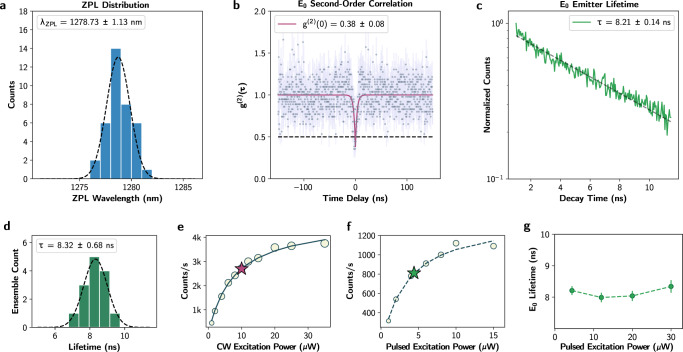
Isolation of single emitters and statistics. **a** ZPL distribution of 37 waveguide-coupled G-centers. **b** Second-order autocorrelation measurement for *E*_0_ shows *g*^(2)^(0) < 0.5 and single photon emission for a continuous-wave excitation power of 10 μW, corresponding to an estimated power density of 5.4 kW cm^−2^. Poissonian error bars are included for each sample point, and error in the *g*^(2)^(0) is the error of the fit. **c** Lifetime of emitter *E*_0_ and **d** statistics for 13 G-center ensembles. Saturation curves under **e** continuous-wave and **f** pulsed excitation fit well to a two-level system. Error bars are denoted by the radii of the circle markers. The excitation power used for measurements (**b**) and (**c**) are marked with purple and green stars respectively. **g** The measured lifetime remains constant under an order of magnitude variation in excitation power, with error bars determined by the error of the extracted lifetime fit.

Figure 3a shows that the ZPL distribution of 37 waveguide-coupled G-centers matches a Gaussian probability distribution with a standard deviation of *σ*_inh_ = 1.1 nm, nearly an order of magnitude narrower than previous reports^8^. Our narrower inhomogeneous distribution may be due to strain relaxation induced by our waveguide geometry (see Supplementary Note 7). The fitted mean ZPL wavelength is 1278.7 nm, in agreement with prior results in bulk SOI^8,27^. Using the number and width of the ZPL peaks in the PL spectrum as an indication of the number of individual emitters within each excitation region, we identified locations within the waveguide that are likely to contain single emitters. We isolated one such area, denoted Emitter 0 (*E*_0_, shown in Fig. 2e), and characterized the photophysics of the artificial atom in this region. The PL spectrum from *E*_0_ is shown in Fig. 1e and exhibits a single resolution-limited peak at 1278.473 ± 0.155 nm. To confirm the presence of a single artificial atom at *E*_0_, we performed second-order autocorrelation (Fig. 3b) and power-dependent emission intensity measurements (Fig. 3c).

### Emitter saturation

Focusing continuous-wave excitation at 532 nm on E_0_, we measured the dependence of the emission count rate on the excitation power. In order to distinguish the contribution to the count rate generated by the emitter from the linear fluorescence observed in the waveguide, we performed background subtraction of the count rate (see Methods and Supplementary Note 5). The background-corrected emission fits well to the characteristic two-level emitter saturation model^27^, given by

1 $$I(P)={I}_{\infty }\frac{P}{P+{P}_{{{{{{{{\rm{sat}}}}}}}}}},$$

with a saturation power ${P}_{{{{{{{{\rm{sat}}}}}}}}}^{{{{{{{{\rm{cw}}}}}}}}}=7.6\pm 0.5\,\mu$W, and a saturation intensity under continuous wave (CW) laser excitation of ${I}_{\infty }^{{{{{{{{\rm{cw}}}}}}}}}=4753\pm 122$ counts per second (Fig. 3e). The extracted saturation power corresponds to an estimated continuous-wave power density of 4.1 kW cm^−2^, assuming a diffraction-limited spot size with objective NA = 0.55. We note, however, that the true excitation area is bounded in one axis by the sub-diffraction waveguide width of 400 nm. These results closely match the saturation power observed for single isolated G-centers in unpatterned SOI wafers^8^. We performed a similar background-subtracted measurement for 532 nm pulsed excitation (see Methods and Supplementary Note 6 for extended details), resulting in a pulsed saturation average power of ${P}_{{{{{{{{\rm{sat}}}}}}}}}^{{{{{{{{\rm{pulsed}}}}}}}}}=3.1\pm 0.4\,\mu$W (Fig. 3f).

### Second-order correlation measurements

Using continuous-wave excitation power of 10 μW (slightly above saturation), we performed second-order autocorrelation measurements using Hanbury-Brown-Twiss interferometry. We split the resulting fiber-collected emission from our sample with a 50:50 O-band fiber beam splitter, followed by detection with two time-tagged SNSPDs (detection efficiencies of 21% and 24% at 1280 nm). We fitted the measured histogram of coincidences as a function of time delay between the detection events to the second-order autocorrelation function of a two-level system emitter, displaying an antibunching dip of *g*^(2)^(0) = 0.38 ± 0.08 (Fig. 3b, see Methods for additional details). The observed antibunching dip with *g*^(2)^(0) < 0.5 confirms single-photon emission and individual addressing of a single artificial atom coupled to a silicon photonic waveguide.

### Emitter lifetime

We subsequently measured the excited-state lifetime statistics of emitter *E*_0_ and 13 other G-center ensembles (a total of 14 spots) using pulsed 532 nm excitation power of 4.4 μW, slightly above the measured pulsed saturation power for a single emitter. Resulting decay curves fit well to a mono-exponential function, following clipping to remove laser leakage and background (see Methods and Supplementary Note 6). The lifetime distribution of the 14 measured G-center ensembles fits well to a Gaussian distribution with mean lifetime 8.33 ns and a standard deviation of 0.68 ns (Fig. 3d). The lifetime of the single G-center at *E*_0_ was measured to be 8.21 ± 0.14 ns (Fig. 3c), in close agreement with the mean lifetimes of the measured ensembles. Additionally, we measured the lifetime of the single emitter *E*_0_ to be constant over an order of magnitude variation in excitation power (Fig. 3g). Our results agree with previously reported lifetimes in bulk SOI G-center ensembles^27^, but indicate shorter lifetimes compared to prior results on isolated G-centers in unpatterned SOI wafers^8^. Calculations of the dipole local density of optical states (LDOS) in our waveguide were compared to the LDOS of a dipole emitter in a SOI slab (see Supplementary Note 2) and suggest that the reduced lifetime we observe is not explained by field enhancement in the patterning alone. Differences in measured lifetimes in our waveguides could be attributed to increased surface recombination in etched surfaces (see Supplementary Note 8). Other effects affecting the measured lifetimes may be doping and defect density variations between wafers. A quantitative characterization of these effects would include geometry-dependent lifetime measurements, which are out of the scope of this study.

### Spectral tuning

**Fig. 4:**
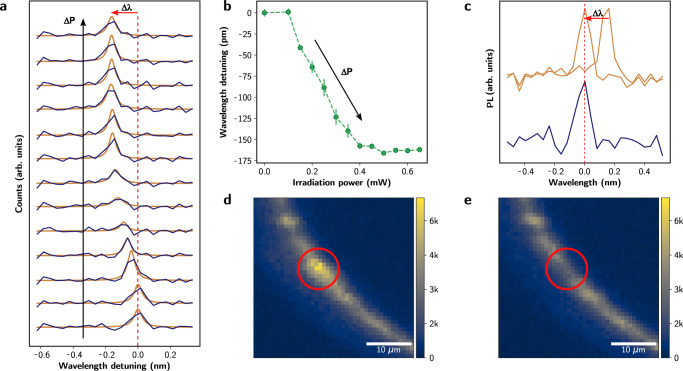
Non-volatile trimming of color centers using light. **a** Example of non-volatile spectral shifting of the ZPL of artificial atoms caused by optical irradiation, with the data represented in blue and the Lorentzian fit in orange. **b** Fitted ZPL central wavelength under increasing optical irradiation power. Error bars denote the error of the Lorentzian fit. **c** Our trimming technique enables non-volatile spectral alignment of separate silicon color centers. PL maps of waveguide sections before (**d**) and after (**e**) local deactivation of an emitter (marked with a red circle).

Moreover, we demonstrate non-volatile spectral trimming and deactivation of single color centers in PICs. Our spectral programming process consists of in-situ local irradiation of G-centers with a 532 nm CW laser with power above 0.1 mW (estimated power density of 54.4 kW cm^−2^) during a duration of 15 s in our 6 K cryostat, followed by probing PL measurements near emitter saturation powers. Under moderate irradiation powers above 0.1 mW, we observe consistent non-volatile spectral shifts of the ZPL for 11 out of our 12 probed emitters. We observe controllable shifts of average amplitude 150 pm (27.5 GHz) and up to 300 pm (55 GHz) as shown in Fig. 4a and b and Supplementary Fig. 10), large enough to match the 25 GHz telecommunication bands and to enable spectral alignment of separate emitters (Fig. 4c). Supplementary Fig. 11a and 11b show the spectral stability of our programmed emitters. Higher powers in the order of 1 mW (estimated power density of 544.3 kW cm^−2^) result in broadening and deactivation of the emitter, leaving the waveguide and adjacent emitters unaffected (Fig. 4d and e and the subtracted PL map in Supplementary Fig. 12). In our experiments, the programming and deactivation effects are non-reversible. Given that our simulations rule out local annealing as a cause for the trimming and deactivation effects (see Supplementary Note 10), we hypothesize that these effects are caused by optically-induced variation of surface charges leading to Stark tuning of the emitters, followed by ionization into the dark A state^25^ for high charge densities. For more information on the process, results, and our hypotheses on the physics behind this effect, see Supplementary Note 10.

## Discussion

The count rates in our waveguide measurements were limited by preventable loss due to imperfect mode matching between our cleaved waveguide facet and the lensed collection fiber. A mode overlap simulation between the SOI waveguide and fiber mode predicts an upper bound on the coupling efficiency in our system to be 8.25% (see Supplementary Note 2). In practice, this excess loss restricts our excitation to a small range near the saturation power of the emitter, as the signal-to-noise ratio of the emission decreases at low excitation powers due to the detector dark counts and also at high excitation powers due to the background fluorescence (see Supplementary Note 5). This low signal to noise ratio is the reason behind our non-zero *g*^(2)^(0). However, the coupling efficiency can be easily improved by up to an order of magnitude using widely available photonic components such as spot-size converters or grating couplers, that can achieve coupling efficiencies in excess of 80%^28,29^.

Another notable difference between our waveguide-integrated G-centers compared to those measured in unpatterned SOI wafers concerns the position of observed emitters selected by the collection mode. Solid-state emitters often show decreased spectral stability in proximity to surfaces due to emitter-surface interactions^30^. Simulations of dipole collection efficiency as a function of dipole depth within our waveguides suggest that we preferentially observe emission from G-centers near the center of the silicon film (Supplementary Note 2, Supplementary Fig. 2b). On the other hand, prior results using confocal collection of G-center emission from silicon slabs select for emitters close to the surface^8^. This geometrical filtering effect may also explain the absence in our measurements of other emitters previously observed in silicon^31^, or those created by etching processes. Our measurements show high emission stability down to a 10 ms timescale for a range of excitation powers, as well as robustness to photobleaching, enabling emission measurements over time intervals greater than 1 month (see Supplementary Note 9). A comparative study looking into G-center emission at varying depths would require probing the emitter-surface interaction for these artificial atoms, but this falls out of the scope of this work.

Our in-situ non-volatile trimming results show a path towards post-fabrication fine tuning of artificial atoms. Such an effect is large enough to tune emitters into standard 25 GHz telecommunication bands, to align emitters to cavities and enhance light-matter interaction, or to spectrally align separate emitters and achieve quantum interference or cooperative emission. In addition, the local deactivation of emitters presented here can aid in trimming waveguides and cavities of unwanted artificial atoms. Further work is required to reveal the physics behind the observed effect and potentially increase its range. This may involve performing higher resolution spectroscopy, measuring samples with different geometries and crystal purity, experimenting with pulsed lasers and other wavelengths, performing in-situ temperature measurements, measuring its dynamics, or searching for correlations between trimming performance and dipole orientation.

Efficient integration with PICs is a key requirement for any large-scale artificial atom qubit platform. Therefore, the results presented here characterizing single G-center emission and trimming in silicon-on-insulator waveguides provide a key step forward for quantum information processing based on color centers in silicon. Moreover, our use of a commercial foundry for the fabrication process makes our results scalable and inherently repeatable by the scientific community, without the need to re-develop an in-house fabrication process. Promising future research directions include the Purcell-enhanced emission from single G-centers in resonant structures aided by localized implantation^32^, and the generation of spectrally indistinguishable single photons for quantum interference using optical trimming, electric fields^33^, or mechanical strain^2^. As demonstrated in other color centers^1^, coupling emitters into cavities helps overcome low quantum efficiencies and Debye-Waller factors. This may not only aid in the development of spin-optic interfaces, but also may enable deterministic single-photon emission and photon-photon interactions via cavity quantum electrodynamics^34^. Investigation of the spin properties of the G-center metastable state^25^, particularly its spin lifetime, and the ^13^C and ^29^Si nuclear degrees of freedom^35^ could additionally enable optically-active quantum memories hosted in silicon photonics. Finally, our results also motivate the study and waveguide integration of other radiation damage centers in silicon, such as the recently isolated T-center^9^ and W-center^7^.

In conclusion, we demonstrated individually addressable artificial atoms operating at telecommunication wavelengths and featuring narrow inhomogeneous distributions in a foundry-written silicon photonic circuit, as well as a method to spectrally program them and deactivate them using light. Our results show native and spectrally programmable single-photon emission and pave the way towards spin qubits in silicon waveguides, establishing silicon photonics as a promising platform for large-scale quantum information technologies.

## Methods

### Sample description

The device under study consists of a background p-doped silicon-on-insulator waveguide with 2 μm silicon dioxide bottom cladding on a silicon substrate. The cross-sectional geometry of the waveguide is shown in Fig. 1f and is rectangular with 400 nm width and 220 nm height. The waveguide starts and ends on a cleaved chip facet and loops in a 63.5 μm radius 180^∘^ bend.

### Sample fabrication

We generated silicon G-centers using a fabrication process that follows^8^. The samples started from a commercial (SOITEC) unclad silicon-on-insulator wafer (220 nm Si on 2000 nm SiO_2_). The wafer was then cleaved into 1 cm^2^ pieces, implanted with ^12^C with a dose of 1 × 10^13^ ions/cm^2^ at 36 keV energy and at an angle of 7^∘^, and then flash annealed for 20 s at 1000 ^∘^C. The sample was then electron-beam patterned and etched in a foundry (Applied Nanotools). The electron-beam patterning was performed using a JEOL JBX8100FS with 100 kV accelerating voltage and positive-tone resist. The silicon etching was performed using inductively coupled plasma reactive ion etching with SF_6_-C_4_F_8_ mixed-gas, in a process optimized for vertical sidewall etching and low propagation loss. This process resulted in silicon waveguides with SiO_2_ bottom cladding and air as top cladding. To enable fiber coupling, the sample was cleaved across the waveguides.

### Photophysics characterization

Optical excitation of the G-centers was performed through the cryostat window using a long working distance objective with numerical aperture of 0.55. Waveguide-coupled emission was collected from a single waveguide output using a SMF-28 lensed fiber and spectrally filtered in free space to select signal between 1250 and 1550 nm. The signal was then detected using either time-tagged SNSPDs or an infrared spectrometer with a wavelength resolution of 155 pm. We measured the excitation powers for all presented measurements immediately prior to the microscope objective.

PL raster scans were acquired by scanning the focused excitation spot over a spatial region of the waveguide sample and gating the SNSPD integration with the electronic trigger of the scanning mirrors. Background-corrected PL spectra were then measured using the infrared spectrometer, where the background spectrum recorded environmental light conditions with the excitation laser blocked.

Saturation curves were measured at a spatial location in the waveguide that exhibited bright PL intensity and a ZPL peak near the 1280 nm transition of the G-center. Correction of the count rate due to the waveguide background was performed prior to fitting with the saturation model in Eq. (1) (see Supplementary Note 5 for details on background measurements and subtraction).

The second-order correlation (*g*^(2)^(*τ*)) was measured with continuous-wave 532 nm excitation near the saturation power. Emission from the waveguide was split with a 50:50 O-band fiber beamsplitter prior to time-tagged detection by DET1 and DET2. A histogram of the coincidences as a function of time difference between detector clicks was acquired with 300 ps binwidth and fitted to the second order correlation of a two-level system^8^:

2 $${g}^{(2)}(t)=b\left(1-(1-a){e}^{-\frac{t}{{\tau }_{1}}}\right),$$

where *a* denotes the normalized zero-delay anti-bunching dip *g*^(2)^(0), *b* describes the background correlation at large time delay, and *τ*_1_ describes the time constant of the zero-delay anti-bunching dip. The resulting second-order correlation of *g*^(2)^(0) = 0.38 ± 0.08 indicates single-photon emission. Poissonian error bars were applied to the data points in Fig. 3b. The error bar on *g*^(2)^(0) refers to the error of the fit calculated from the covariance in Python’s scipy.curve_fit() function.

Emitter decay lifetimes were measured using pulsed 532 nm excitation at a repetition rate of 34 MHz. A histogram of the SNSPD clicks as a function of the delay time between the detection event and the pulsed laser trigger were first clipped to remove laser leakage and background (see Supplementary Fig. 6a, b for details). The resulting decay curves fit well to a mono-exponential function:

3 $$f(t)=c{e}^{-\frac{t-{t}_{0}}{\tau }}$$

with an extracted emitter lifetime of *τ* = 8.21 ± 0.14 ns. Here, *c* describes a normalization factor given by the number of detected counts at the pulse peak, *t*_0_ is a time shift of the peak, and the error bar on the lifetime of a single emitter is again calculated from the error of the fit. The emitter characterization and spectral tuning data presented in this study are available in the Zenodo database under 10.5281/zenodo.7679379.

### System efficiency

The collection efficiency of our system was characterized as follows:

- *η*_ec_: Simulated edge coupling efficiency between the cleaved waveguide mode and a perfectly aligned lensed fiber was calculated to be 8.3% at 1280 nm.
- *η*_dipole−wg_: Maximum dipole coupling to a uni-directional waveguide mode was simulated to be 40% (see Supplementary Fig. 2a and b for details).
- *η*_filt_: Free space fiber filtering with two fiber collimators was measured to have 51.3% transmission at 1280 nm.
- *η*_fiber_: Fiber routing transmission to the two SNSPD cryostat detectors DET1 and DET2 was measured to be 90.6% and 94.8%, respectively.
- *η*_splitter_: Insertion loss of fiber beamsplitter was measured to be 92%.
- ${{{{{{{\eta }}}}}}}_{\det }$: SNSPD detector efficiencies at 1280 nm for DET1 and DET2 were characterized to be 24% and 21%, respectively. These SNSPDs were manufactured to have high efficiency at a target wavelength of 1550 nm.

The total system efficiency, given by

4 $${\eta }_{{{{{{{{\rm{tot}}}}}}}}}={\eta }_{{{{{{{{\rm{ec}}}}}}}}}{\eta }_{{{{{{{{\rm{filt}}}}}}}}}{\eta }_{{{{{{{{\rm{fiber}}}}}}}}}{\eta }_{{{{{{{{\rm{splitter}}}}}}}}}{\eta }_{\det }$$

can, therefore, be upper bounded to 0.34% for DET1 and 0.31% for DET2. Using the above estimates for the collection efficiency and an observed saturated count rate of 1382 ± 59 counts/second under 78 MHz pulsed excitation, we estimate a lower bound on the quantum efficiency of 1%. However, the uncertainty on this estimate is large, due to low measured count rates and estimates of *η*_ec_ and *η*_dipole−wg_ being derived from simulation.

## Supplementary information

Supplementary Information

## Data Availability

The emitter characterization and spectral tuning data presented in this study are available in the Zenodo database under 10.5281/zenodo.7679379.
