# Supplementary material for: Individually addressable and spectrally programmable artificial atoms in silicon photonics
Source: Nat Commun. 2023 Apr 25;14:2380. doi: 10.1038/s41467-023-37655-x (PMC10130169; doi:10.1038/s41467-023-37655-x)
Supplement: Supplementary file 1 — Supplementary Information [file 41467_2023_37655_MOESM1_ESM.pdf]

Supplementary Information: Individually Addressable and Spectrally Programmable Artificial Atoms in Silicon Photonics

Mihika Prabhu[1,†,*], Carlos Errando-Herranz[1,2,†,*], Lorenzo De Santis[1,3],
Ian Christen[1], Changchen Chen[1], Connor Gerlach[1], and Dirk Englund[1,*]
[1] *Massachusetts Institute of Technology, Cambridge, USA*
[2] *University of Münster, Münster, Germany*
[3] *QuTech, Delft University of Technology, Delft, Netherlands*
[†] *Equal contribution*
* *mihika@mit.edu, carloseh@mit.edu, englund@mit.edu*
(Dated: March 25, 2023)

SUPPLEMENTARY NOTE 1 - MEASUREMENT SETUP

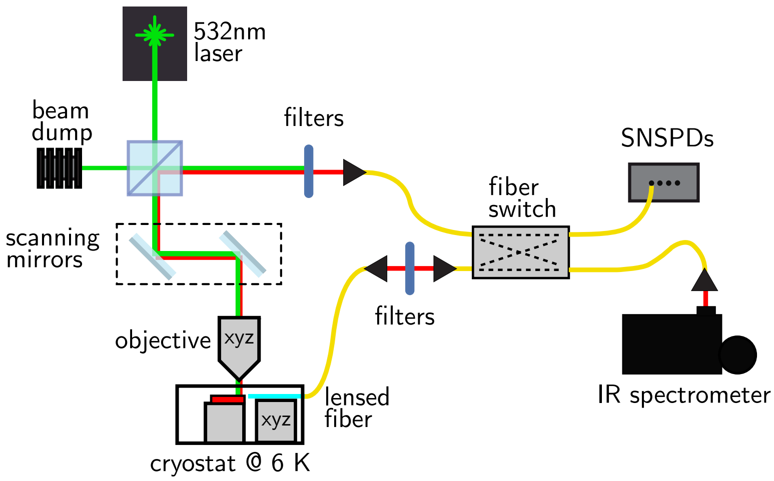

Supplementary Figure 1. **Measurement setup schematic.** Our setup combines confocal microscopy with fiber coupling. Laser light shines through a beam splitter and a set of galvanometers into an objective. For fiber-coupling, the PL from the waveguide couples into an aligned lensed fiber via free-space filters into a fiber switch leading to SNSPDs or an IR spectrometer. For confocal microscopy, PL from the sample reflects off the beam splitter via a filtering stage into the fiber switch.

Supplementary Figure 1 shows the measurement setup we used in this work. Description of the components of the measurement apparatus pertaining to the optical characterization of emitters is presented in the Methods section of the main text. We include additional setup details in this section for completeness. Our continuous wave excitation source is a Coherent Verdi G5 at 532 nm, and our pulsed excitation is an NKT Photonics SuperK laser with a maximum repetition rate of 78 MHz and a pulse length below 1 ns. To avoid background from our laser, the excitation path was filtered with a 532 nm band-pass filter (Semrock Maxline bandpass with 2 nm FWHM). Our microscope objective is a Mitutoyo 50× M Plan APO and Mitutoyo 100× M Plan APO SL.

Our cryostat is a Montana Instruments CR-057 with a home-made fiber feedthrough. The fiber we use (OZ Optics, spot size of 2.5 µm at 1550 nm, and expected spot size of 2.1 µm at 1300 nm) is mounted on a XYZ cryogenic piezoelectric stage (Attocube). Our free-space filtering setup consists of a 1250 nm longpass (Thorlabs FEL1250) and a 1550 nm shortpass (Edmund Optics OD2) filters. To detect our PL we use two superconducting nanowire single photon detectors (NIST) with detection efficiencies 21% and 24% and timing jitters below 172 and 165 ps, readout with a Swabian Instruments Timetagger 20. Alternatively, we use an IR spectrometer consisting of a PyLon IR InGaAs linear CCD array from Princeton Instruments and two gratings with densities of 300 g/mm and 900 g/mm, 1.2 µm and 1.3 µm blazes, and resulting resolutions of 155 pm and 40 pm respectively. For the second order autocorrelation measurement we used a fiber beam splitter (Thorlabs TW1300R5F1) after the filtering station.

SUPPLEMENTARY NOTE 2 - COUPLING EFFICIENCY SIMULATIONS BETWEEN EMITTER, WAVEGUIDE, AND FIBER

Finite difference time domain (Lumerical FDTD) simulations were performed to calculate the emitter-waveguide coupling as a function of the emitter height in the waveguide. Directional waveguide-coupled dipole emission was measured using a power monitor at one end of the waveguide, with a dipole emitter oriented in either the X-, Y-, or Z-direction positioned at a range of heights within the waveguide (Fig. 2a). The single-ended waveguide transmission for each direction and dipole height is shown in Fig. 2b. We observe an improved waveguide coupling for dipoles oriented transverse to the waveguide axis and near the center of the silicon film. Our asymmetric waveguide geometry preferentially supports quasi-TE modes over quasi-TM, so the improved coupling for the dipoles oriented along the long transverse axis of the waveguide agrees with expectations. We

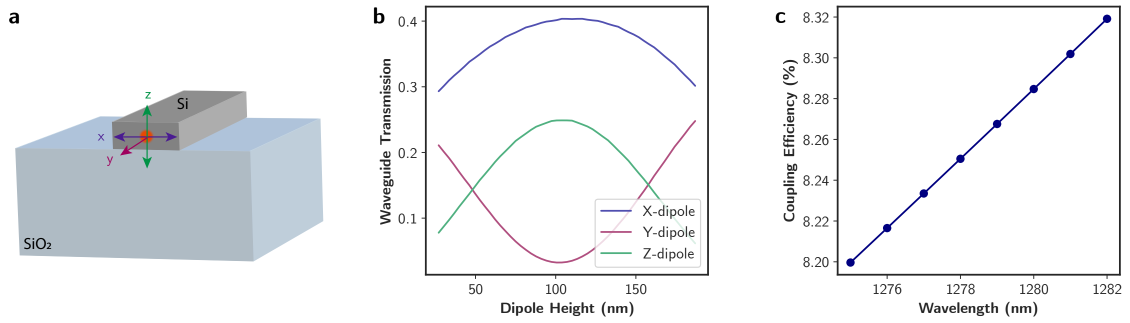

Supplementary Figure 2. **Coupling efficiency simulations.** a) Schematic showing the geometry and the dipole orientations simulated for b) the uni-directional waveguide coupling results. c) Coupling efficiency simulated via modal overlap between our lensed fiber mode and our cleaved waveguide facet.

note that the waveguide collection could be improved by up to a factor of two by adding a reflector at the other end of the waveguide.

The edge coupling efficiency between the cleaved waveguide mode (shown in Figure 1f) and a perfectly aligned lensed fiber was simulated using Lumerical MODE Solutions eigenmode solver. We simulated the mode at the focus spot of the fiber as a Gaussian beam with 2.1 µm $1/e^2$ diameter, and the waveguide mode as the output of a waveguide mode eigenmode simulation. Our mode overlap calculations, including the Fresnel reflection at the waveguide-air interface, yield the coupling efficiency in Fig. 2c, and a 8.25% efficiency at 1280 nm.

SUPPLEMENTARY NOTE 3 - CONFOCAL COLLECTION FROM VERTICALLY-COUPLED STRUCTURES

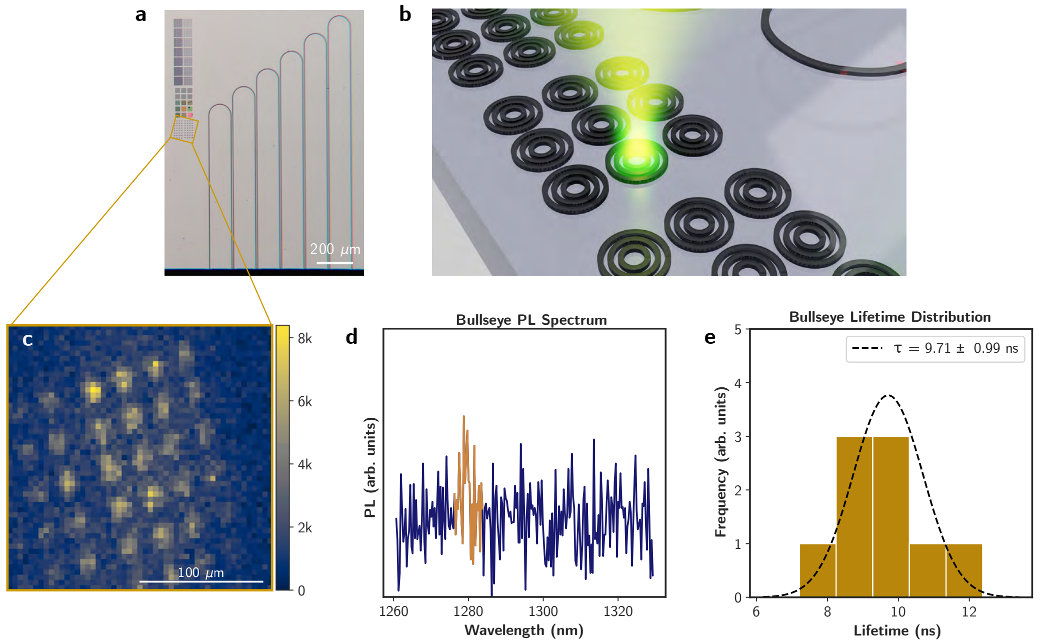

Supplementary Figure 3. **Confocal collection of G-centers from circular grating bullseye structures at 6 K.** a) A sample micrograph and b) a schematic of the bullseye structures used to enhance vertical coupling of G-centers. c) The PL map shows emission from the structures. d) PL signal from one of the devices. e) Lifetime statistics for 9 devices.

In addition to waveguides, the fabricated G-center sample consists of bulls-eye grating structures, designed

for a vertically-coupled cavity mode near 1275 nm (Fig. 3a-b) with $Q \sim 500$. The objective used for both excitation and collection was a Mitutoyo 50× M Plan APO long working distance objective (NA=0.55). It is worth noting that the specified objective has a designed operating range of 435-655 nm, which is optimized for transmission of the 532 nm excitation path. However, confocal collection around 1280 nm falls outside this range. Raster scans of the vertically-coupled PL signal were acquired in the same way as the through-waveguide measurements. Distinct PL intensity maxima are observed over the bulls-eye structures (Fig. 3c); however, the observed count rates were much lower for the same excitation power than with through-waveguide emission. A background-corrected PL spectrum from one of these structures was collected with the maximum spectrometer integration time of 800 seconds. A dim peak at 1278.622 nm was observed (Fig. 3d), suggesting the presence of the G-centers in these structures. Lifetimes for 9 of these structures were measured, using the same protocol for fitting as the waveguide samples. The distribution of the lifetimes in the bulls-eyes was observed to be 9.71 ± 0.99 ns, slightly higher than those observed for G-centers in waveguides.

SUPPLEMENTARY NOTE 4 - ION IMPLANTATION SIMULATIONS

The carbon ion implantation depth was simulated using Stopping and Range of Ions in Matter (SRIM).The simulated layer was 220 nm of ^{28}Si with a density of 2.3212 g/cm^3, and the simulated ions were ^{12}C with an acceleration of 36 keV and incident at an angle of 7°. The simulation results, shown in Fig. 4 yield an ion range (i.e. mean ion depth) of 113.3 nm with a straggle (i.e. standard deviation) of 41.3 nm, a skewness of -0.259 and a Kurtosis of 2.6411.

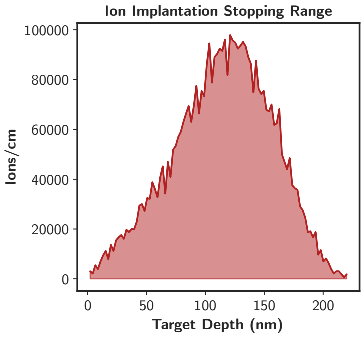

Supplementary Figure 4. **Ion implantation depth simulation.** A simulated mean ion depth of 113.3 nm and a standard deviation of 41.3 nm are expected.

SUPPLEMENTARY NOTE 5 - BACKGROUND CORRECTION FOR SATURATION

A PL signal was observed in the waveguide regions between PL maxima, particularly at high excitation powers. As a result, a protocol to measure and subtract this background was necessary to separate the count rate from the emitters from the background counts generated in the waveguide. First, PL maps were taken to isolate points that contain G-centers, as well as to locate a point in the waveguide to measure the background. The regions isolated for this measurement are shown in Fig. 5a. The spectra of the PL signal from the two emitter points, E_0 and E_1 show ZPL peaks near 1280 nm, whereas the background waveguide region shows no ZPL emission (Fig. 5b). The count rate at the single emitter spot E_0 and the background region was measured over a range of excitation powers. The background-corrected emitter saturation was calculated by subtracting the total count rate at the emitter from the counts measured in the waveguide background region. Supplementary Figures 5c-d show the results of the background correction for both CW and pulsed 532 nm excitation.

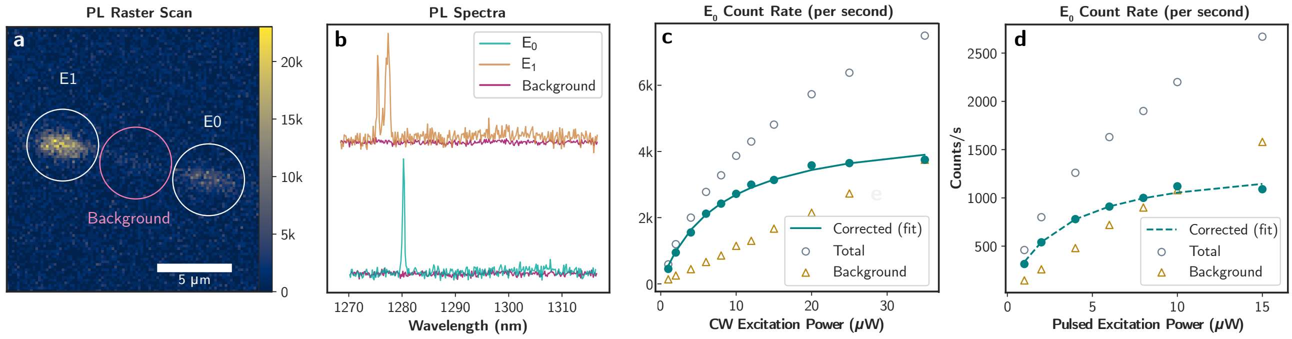

Supplementary Figure 5. **Background subtraction protocol for G-center through-waveguide measurements at 6 K.** a) A PL raster scan showing emitter and background regions of the waveguide. b) PL spectra for the three regions indicated in (a). c) Background-subtracted saturation curve for CW 532 nm excitation. d) Background-subtracted saturation curve for pulsed 532 nm excitation.

SUPPLEMENTARY NOTE 6 - LIFETIME FITTING

The emitter lifetimes were measured using 532nm pulsed excitation from the SuperK laser. A repetition rate of 34 MHz was selected to enable decay curves of up to 29 ns to be acquired. The resulting raw decay curves exhibit both an initial peak at the onset of the pulse, as well as flattening towards the end of the pulse period from count background. Fig. 6a shows a plot of the normalized emitter lifetime compared to the normalized lifetime of the pulsed laser. A bi-exponential fit of the raw lifetime data confirms that the time constant of the initial peak matches with that of the excitation laser. Additionally, it can be seen in Fig. 6b that increasing the excitation power does not change the lifetime slope; however, the fractional contribution of the initial peak to the total counts increases with higher laser power. Furthermore, it is observed that the decay becomes background-limited near 15 ns post-excitation. The final lifetime is calculated by clipping the raw lifetime to a range between 1 ns and 12.5 ns before fitting to a single exponential function (Fig. 6b).

We compared our simulations of the local density of optical states (LDOS) for a dipole emitter in our waveguide (400 nm $\times$ 220 nm) to those of the dipole LDOS in a 220 nm thick slab SOI to estimate the change in the radiative lifetime of our patterned sample. The dipole radiation power was simulated in Lumerical FDTD along the three cardinal axes to calculate the total LDOS for an emitter with a random orientation within the waveguide. A similar simulation was calculated for dipoles placed in a slab SOI. The radiative lifetime, τ, is inversely proportional to the LDOS, ρ, so the suppression in radiative lifetime in the waveguide compared to a slab is

$$\frac{\tau_{\text{wg}}}{\tau_{\text{slab}}} = \frac{\rho_{\text{slab}}}{\rho_{\text{wg}}} \tag{1}$$

and was calculated over a range of possible dipole heights within the waveguide (Fig. 6c). From this simulation, we observe that the change in radiative lifetime due to waveguide patterning of a slab does not fully explain the difference observed between our results and that of isolated single G-centers in SOI slabs [1].

SUPPLEMENTARY NOTE 7 - SIMULATIONS OF STRAIN DISTRIBUTION IN SOI WAVEGUIDES AND SLABS

It is well known that commercial SOI wafers have significant intrinsic stress. The presence of a defect in the lattice induces stress relaxation into strain gradients localized around the defect, which may result in a significant inhomogeneous ditribution for a constrained SOI slab, such as for the results presented in [1]. In contrast, for the more mechanically compliant case of a patterned waveguide, the stress in the film relaxes largely into homogeneous strain (i.e. by deforming the waveguide cross-section), potentially resulting in a narrower inhomogeneous distribution.

We qualitatively modeled this effect by assuming a nanoscale air defect (simulated as an air gap of 1 nm radius) in a isotropic silicon film. Using COMSOL Multiphysics v5.5, we simulated a 2D silicon slab with fully clamped sidewalls and our defect under the 38 MPa biaxial (x-axis in the simulation) compressive stress reported

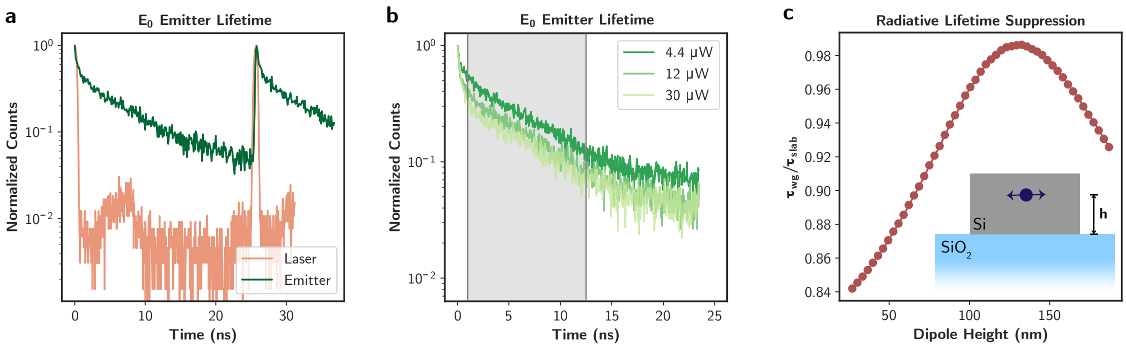

Supplementary Figure 6. **Emitter lifetime fitting and simulations.** a) Lifetime measurement comparative between an emitter and the green laser, highlighting the need to time-clip the signal. b) The emitter lifetime measurements under varying excitation power yield negligible variation for an order of magnitude power variation. c) Our simulations of Purcell enhancement in a waveguide versus dipole height in a SOI slab yield a nearly-negligible effect on the expected lifetime ratio. The measurements in a) and b) were performed at 6 K.

for photonic SOI.The resulting strain distribution is shown in Fig. 7a and b, and shows height-independent volumetric strain gradients in the order of $\varepsilon_{\text{max}} = 20 \times 10^{-5}$ in the vicinity of the defect. In contrast, the simulation results for a 400 nm wide waveguide in Fig. 7c and d show reduced strain gradients for all defect positions ($\varepsilon_{\text{max}} = 10 \times 10^{-5}$) and an order of magnitude ($\varepsilon_{\text{max}} = 1.5 \times 10^{-5}$) in the center of the waveguide.

Although these results do not quantitatively model a real defect in a solid, they provide a qualitative intuition for a possible explanation to our observation of a reduced inhomogeneous distribution in waveguides.

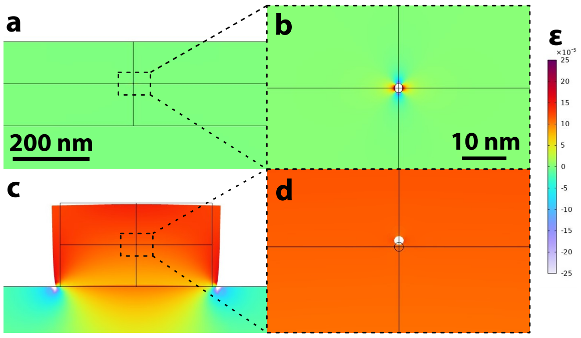

Supplementary Figure 7. **Simulations of strain distribution for a slab and a waveguide.** a) The strain distribution in a SOI slab. b) The zoom-in shows large strain gradients concentrated around the defect. c) The waveguide-patterned structure relaxes its stress by strain. d) The zoom-in around the defect shows up to an order of magnitude reduced strain gradients.

SUPPLEMENTARY NOTE 8 - DEFECT-RELATED CARRIER DYNAMICS

The photodynamics of the G-center emission under above-bandgap excitation is described here with the rate equation model depicted in Fig. 8. As silicon possesses an indirect bandgap at a transition near 1100 nm (1.17 eV), we assume that the band-to-band radiative recombination rate is negligible. The primary mechanisms governing carrier dynamics in silicon are Shockley-Read-Hall (SRH) trap recombination, surface recombination, and Auger recombination [2]. The waveguide devices presented in this paper do not have a top oxide cladding and are not passivated to reduce surface trap states; therefore, we expect a non-negligible recombination from surface effects at the waveguide sidewalls. The main pathway to populate the excited state of the G-center is determined by the rate γ_{42}, which is an effective rate including the influence of trap states in silicon. Once populated, the emission dynamics from the G-centers is following the behaviour of a 2 level system with an

additional shelving state.

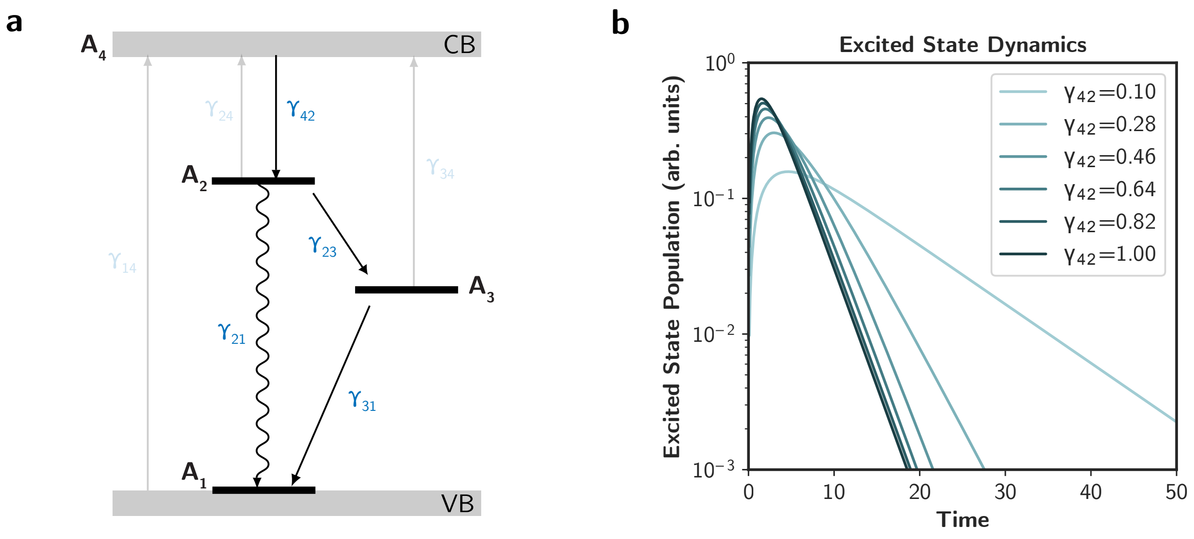

Supplementary Figure 8. **Energy level description and rates.** a) Energy levels and transitions for defect-assisted recombination in G-centers. A_4=CB=conduction band, VB=valence band, A_2-A_1 is the G-center ZPL transition, and A_3 is the metastable state. Note that here we made A_1 degenerate with VB as described in Ref. [3]. b) Excited state dynamics show a decrease in lifetime under increased electron capture rates γ_{42}.

The dynamics of this system are described by the following rate equations, where N_i corresponds to the carrier occupation density at level A_i.

$$\frac{d}{dt}\begin{bmatrix} N_1 \\ N_2 \\ N_3 \\ N_4 \end{bmatrix} = \begin{bmatrix} -\gamma_{14} & \gamma_{21} & \gamma_{31} & 0 \\ 0 & -(\gamma_{24}+\gamma_{23}+\gamma_{21}) & \gamma_{32} & \gamma_{42} \\ 0 & \gamma_{23} & -(\gamma_{31}+\gamma_{32}+\gamma_{34}) & 0 \\ \gamma_{14} & \gamma_{24} & \gamma_{34} & -\gamma_{42} \end{bmatrix} \begin{bmatrix} N_1 \\ N_2 \\ N_3 \\ N_4 \end{bmatrix} \tag{2}$$

The transition rates γ_{ij} from state A_i to state A_j depend on a number of material properties in the patterned silicon structures, and as such, the calculation of the exact rates is out the scope of this study. The level occupation densities can be obtained using a matrix exponential solution to the system of coupled differential equations. Due to the coupled nature of the rate equations, the time-dependent state occupations depend on contributions from each of the rate terms, determined by the eigenvalues of the coefficient matrix in Equation (2). The assumptions and general physical mechanisms that can govern these rates are described below:

- γ_{14}: denotes the electron pumping rate, under the assumption that the conduction band occupation is much lower than the valence band occupation ($N_1 >> N_4$). Our lifetime measurements are performed under pulsed excitation, so we assume pumping and re-pumping processes (including γ_{24} and γ_{34}) to the conduction band to be zero after the initial pulse.

- γ_{24}: the rate of thermalized excitation of electrons from the upper defect level to the conduction band (a function of material doping and temperature, T), which includes a re-pumping term (set to zero after the pulse, see above).

- $\gamma_{42} \propto \sigma_e N_{\text{trap}} N_{\text{carrier}}$: the rate of electron capture from the conduction band to the upper trap state depends on material properties of the wafer and structures, including the electron capture cross-section, σ_e; the density of traps, N_{trap}; and the carrier density, N_{carrier}.

- $\gamma_{21} \propto \frac{F_{\text{Purcell}}}{\tau_{\text{rad}}}$: describes the effective radiative emission rate from the defect excited state to ground state in the presence of a perturbed local density of optical states (LDOS), where enhancement in the spontaneous emission rate is quantified by the Purcell factor.

After laser excitation, we assume all the carrier population in N_4 (i.e. the conduction band) and let the system of equations evolve over time. Supplementary Figure 8b shows the evolution of the carrier density in the excited state A_2 in this model while varying the rate of electron capture to our defect, γ_{42}. We observe that an increased γ_{42} leads to shorter lifetimes.

The measured G-center lifetimes reported in the literature vary between 4.5 ns [4, 5] and 35.8 ns [1]. There are many possible physical mechanisms that can result in variations in the carrier dynamics, including band bending from surface charges or from compressive strain in the silicon wafer, wafer-to-wafer variations in silicon thickness and doping, changes to the electron capture cross-section or carrier density due to the presence of surface states, or opportunistic emission from other defect centers. Correcting for these sample-dependent variations, perhaps by using techniques such as surface passivation using atomic layer deposition, will be a critical step towards building a scalable artificial atom platform in SOI. However, investigation of these mechanisms is out of the scope of the current study.

SUPPLEMENTARY NOTE 9 - PL STABILITY MEASUREMENTS

We investigated the stability of our waveguide-coupled G-centers via time-tagged PL collection. Supplementary Figure 9a shows a time trace of PL emission from a single G-center during a 100 s acquisition period with a binning time of 10 ms. Supplementary Figure 9b shows comparative histograms for the emitter and the waveguide background under a range of excitation powers. Our emitter histograms fit to Poisson distributions, suggesting non-blinking and stable behavior at timescales longer than 10 ms. Finally, Supplementary Figure 9c shows that spectral resolution-limited PL can be observed from a single emitter in measurements separated by over a month and following multiple cryostat cooldown cycles. The spectral shift in the ZPL peak between the two measurements is likely caused by the re-calibration of the spectrometer.

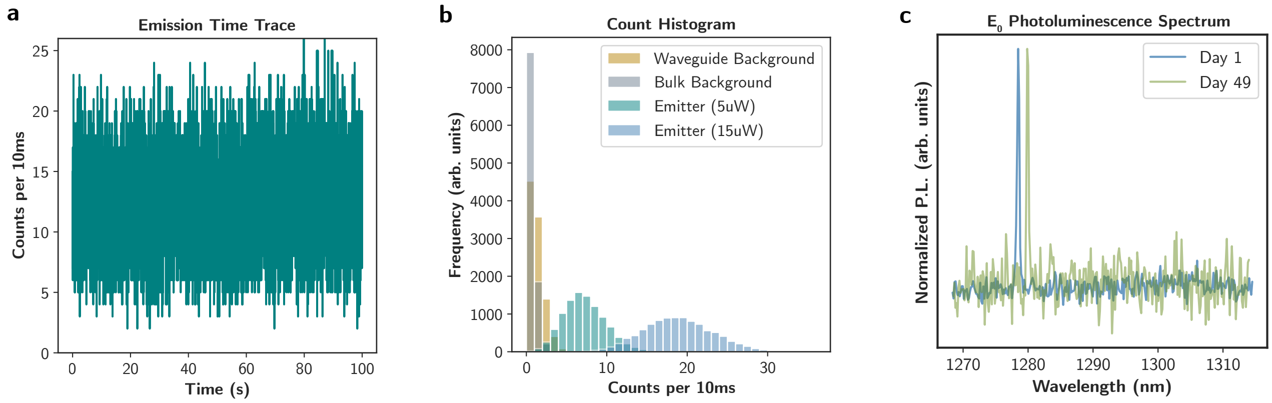

Supplementary Figure 9. **PL emission stability measurements at 6 K**. a) Time trace showing stable PL emission from a single G-center for 100 seconds at 10 µW 532nm continuous-wave excitation. b) The comparative histograms show that the frequency of counts for a 10 ms time bin width at 5 µW excitation fits a Poisson distribution. c) Normalized photoluminescence spectra collected from a single emitter with acquisition separated by 49 days. Note that the apparent ZPL wavelength shift is largely caused by the re-calibration of our spectrometer in between those measurements.

Our stability measurements are limited to a 10 ms time bin due to our limited counts arising from our low system efficiency. Higher coupling efficiencies are required to probe emitter stability at faster timescales.

SUPPLEMENTARY NOTE 10 - OPTICAL TRIMMING OF SILICON COLOR CENTERS

Our trimming experiments were performed in-situ in our cryogenic setup described in Supplementary Note 1 without additional modifications. Our trimming protocol consisted of:

1. Localization of emitters by PL maps using 25 µW (estimated power density of 13.6 kW·cm^{-2}) of 532 nm CW laser excitation and our SNSPD detectors, followed by spectrometry (900 gr/mm grating, 40 pm resolution) under the same excitation conditions.

2. Local irradiation with a 532 nm CW laser with powers in the order of 100 µW (estimated power density of 54.4 kW·cm^{-2}) for 15 s.

3. Characterization of the emitter by PL maps and spectrometry as in the first step.

4. Repetition of steps 2-3 with increasing irradiation power.

We performed our trimming characterization protocol on 12 bright emitters (5000 to 10000 cts/s on our SNSPDs with a 1300-1550 nm bandpass filter) under PL excitation. From those, we observe trimming in 11 emitters, with spectral measurements and Lorentzian fits shown in Figure 4 and Supplementary Figure 11b and c. We observe spectral blue shifts in 9 out of the 11 shifting emitters, while two of them red shift, including emitters in the same spatial location shifting in opposite directions. The maximum magnitude of the trimming is 300 pm (55 GHz) and depends on the emitter, with a relatively constant bandwidth limited by our spectrometer resolution. When nearing 1 mW of irradiation (estimated power density of 544.3 kW·cm^{-2}), some of the emitters appear to shift back to near their original spectral position. Consecutive measurements without irradiation at any step in the process did not show spectral shift (see Fig. 11a and b for continuous monitoring of a small ensemble over 40 min). The emitter that did not show shifts showed instead diminishing brightness with increasing irradiation power, and is plotted in Fig. 11c and d. We note that we did not observe an increase in the cryostat temperature during our measurements.

For higher powers, we observe local deactivation of emitters. Figure 4d shows a PL map of a section of our waveguide containing several emitters, with a brighter ensemble in the center where the irradiation was focused. For this location, after 0.9 mW (estimated power density of 489.9 kW·cm^{-2}) of local irradiation for 60 s, the small ensemble in the center is deactivated, as shown in Figure 4e, without deactivating nearby emitters. This is evidenced by the subtracted PL map in Fig. 12, showing the location where the emitter was deactivated. The faint mark following the waveguide in Fig. 12 is a result of a slight misalignment between the first and second PL maps.

Local annealing of point defects in the waveguide could explain these observations. To explore this hypothesis, we simulate the thermal performance of our waveguide system subjected to localized heating. We assume 100 µW of heat incident upon a 300 nm×300 nm square at the top of the waveguide. This corresponds to a power on the order of 1 mW of 532 nm excitation: roughly a third of this power is reflected by the silicon-air interface; a tenth is assumed to be absorbed by the waveguide section; and the remainder is transmitted through the waveguide and reflected and absorbed in the silicon substrate or elsewhere along the waveguide. The assumed absorption is estimated from silicon's room-temperature optical attenuation coefficient of $\sim 10^4$ cm^{-1} at 532 nm [6]; there is evidence that the absorption coefficient at telecommunication wavelengths is roughly constant down to low temperatures [7]. We use thermal conductivity estimates at 5 K for thin silicon (10 W/m/K) and silicon dioxide (0.1 W/m/K) from Ref. [8] and the same for the bulk silicon (100 W/m/K) substrate from Ref. [9]. Note that these thermal conductivities increase with temperature for the temperature ranges considered, so our simulation with fixed conductivities overestimates the achieved temperature. Based on our simulations, we estimate an upper bound maximum temperature of 200 K at the laser spot with a full-width-half-maximum of roughly 5 µm (Fig. 13). Since these centers are stable up to room temperature and stand flash anneals at 1000°C, the hypothesis of local annealing is thus inconsistent with our observations.

Our leading hypothesis is that the spectral shifts observed stem from DC Stark tuning arising from optically-induced charge variations, and that deactivation stems from ionization into the dark charged state of the G-center [3]. Photoinduced spectral shifts have been previously reported in diamond color centers, and have been associated with trapped charges in the lattice [10]. It is well known that Si/SiO_2 interfaces contain a large density of hole traps [11] with an experimentally-measured saturation at densities in the order of $\sigma_h = 1\times10^{13}$ cm^{-2} [12]. We hypothesize two flavors of this effect depending on initial conditions: 1) in the presence of existing charges, optical excitation may reduce the magnitude of the charge experienced by the emitter via recombination, and 2) in the absence of charges, carrier generation may result in higher densities of trapped holes. In both cases, the relative charge difference is the same, and thus in the following we estimate the effect that such charge variation can have on an artificial atom via DC Stark tuning. Given the large difference in area between our single emitters and the waveguide surface, we can assume an infinitely large charged sheet, with an associated external electric field of $E = \frac{\sigma}{2\epsilon}$. σ is the charge density, which for SiO_2 is in the order of $e\sigma_h$, with e the elementary charge. This estimate leads to electric fields in the order of 100 MV/m. Assuming DC Stark tuning rates in the order of $R = 10$ GHz/MV m, in line with other color centers such as NV in diamond [10], we estimate maximum spectral shifts in the order of $\Delta\lambda_0 = R \times E = 1000$ GHz. We note that our estimated maximum spectral shift will be significantly affected by our device geometry and oxide interface quality (likely different between the native oxide and thermally-grown oxide at the top and bottom of our waveguide), our

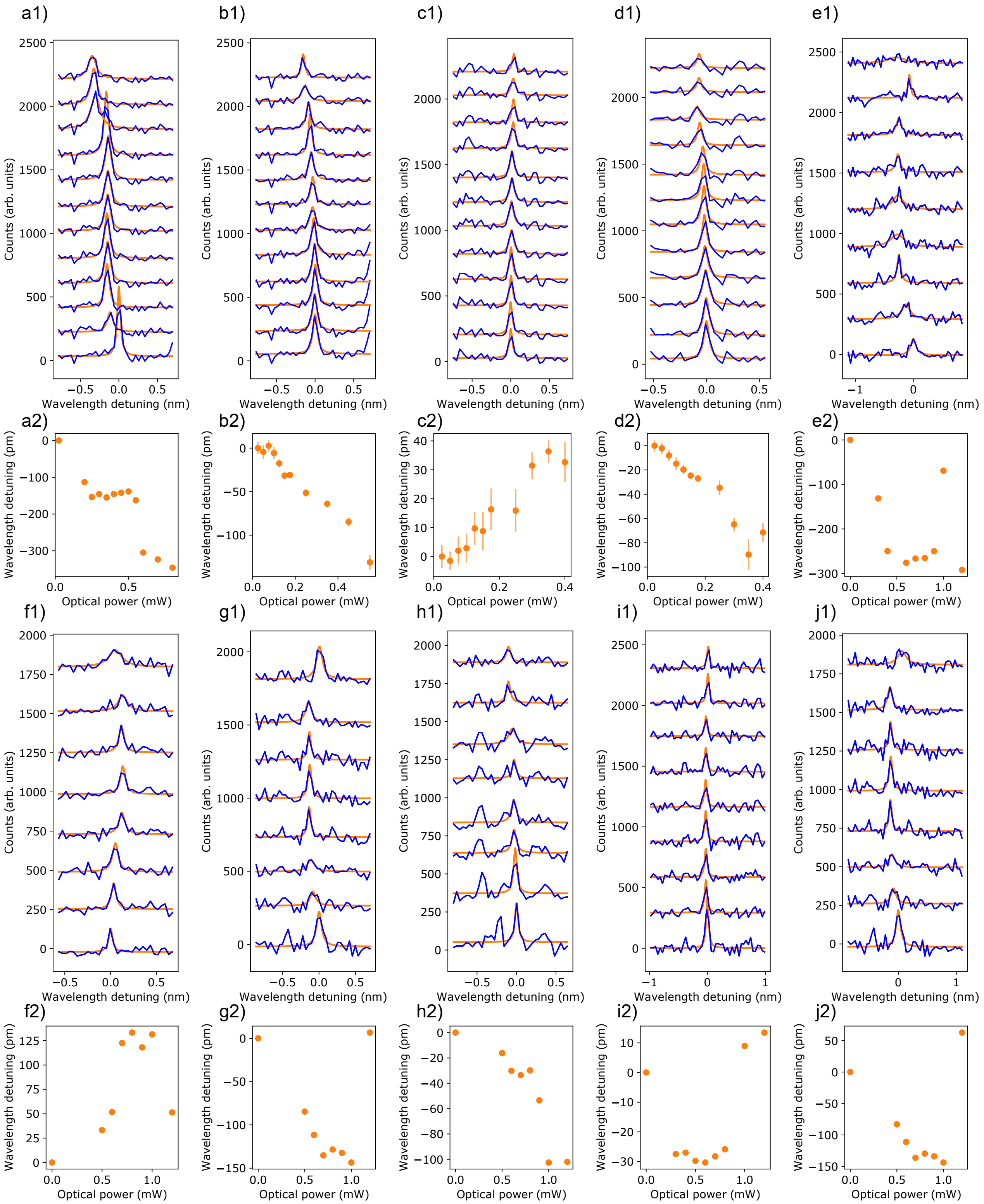

Supplementary Figure 10. **Trimming results for all the tested devices at 6 K.** Each vertically stacked pair of subfigures (e.g. a1) and a2)) are from the same emitter. 1) Spectra under increasing irradiation power (blue, increasing power from bottom to top within each subfigure), and Lorentzian fitting (orange). 2) Fitted central wavelength shift versus irradiated optical power. All the probing measurements were performed with 10 µW of excitation power.

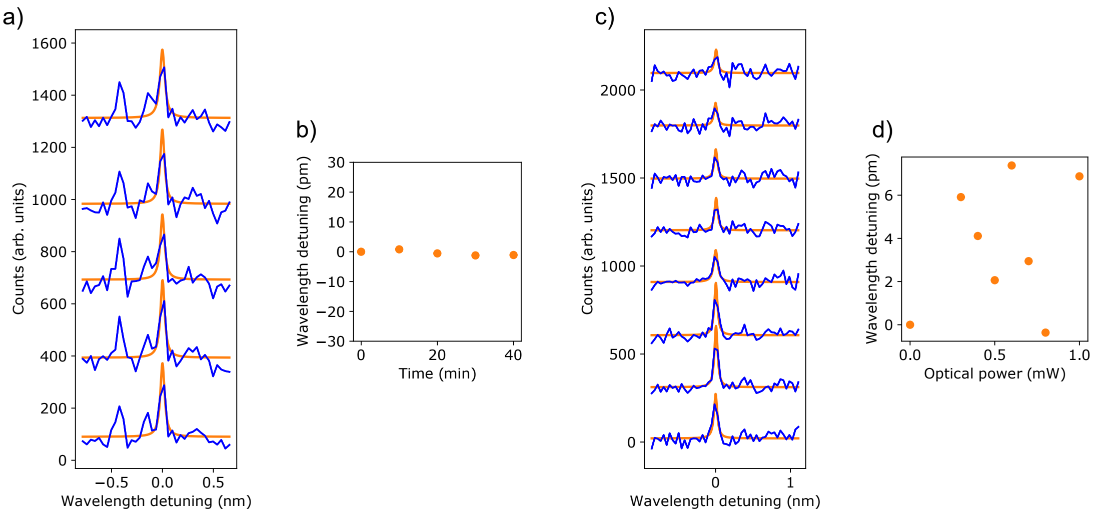

Supplementary Figure 11. **Stability of trimmed emitters at 6 K.** a) The stability of the PL spectra of a small ensemble of emitters (where b) shows a fit to one of the emitters) monitored after trimming demonstrates the non-volatility of our method. c) Fitted spectra and d) central wavelength for the only non-trimmable emitter within a sample of 12. All the probing measurements were performed with 10 µW of excitation power.

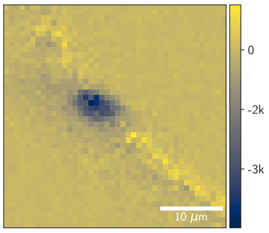

Supplementary Figure 12. **Deactivation of emitters with high optical powers.** The subtracted PL map from Figures 4d and 4e show localized deactivation of single emitters and small clusters.

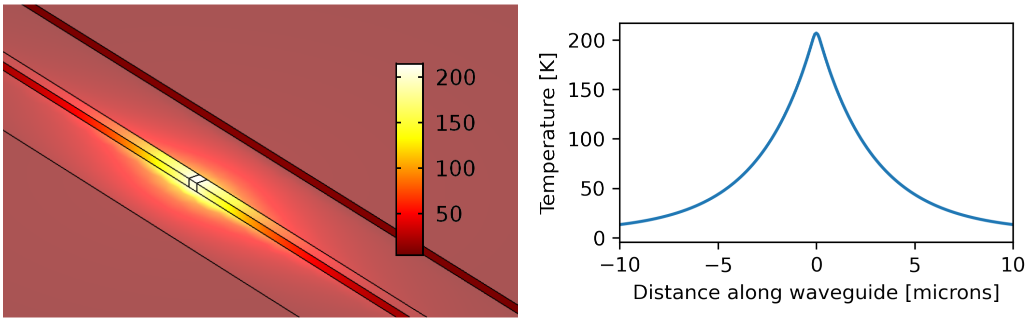

Supplementary Figure 13. **Simulation of local temperature increase in an irradiated waveguide.** A 3D plot of the simulated system (left), including the Si waveguide and SiO_2 bottom cladding. The color bar represents temperature in K. The white cube in the center acts as the heat source in our simulation. A 1D slice along the center of the waveguide (right) shows a full-width-half-maximum of ∼5 µm.

estimate of Stark tuning rate, or other potentially more stringent limits such as defect ionization or charge recombination effects. For high irradiation powers, one explanation is that ionization takes place, similarly to the previously reported photoionization of the NV color center in diamond [13].

We would not like to rule out a second hypothesis, in which optical irradiation breaks Si-Si bonds resulting

in an excess of dangling bonds, which then shift the artificial atom spectral lines via a strain or electric field redistribution. Such an effect was first reported by Staebler and Wronski [14], by noting that light affects the conductivity of amorphous silicon via metastable defects. The cause was later attributed to the creation of dangling bonds by Stutzmann et al. [15].

SUPPLEMENTARY NOTE 11 - SECOND-ORDER CORRELATION WITH A THREE-LEVEL ATOM MODEL

The electronic level structure of the G-center defect has long been reported to consist of two singlet states and a metastable triplet state [3, 16]. The signature bunching effect in the second-order correlation, characteristic of a recombination process mediated by a three-level system, has been experimentally observed for G-centers in bulk SOI wafers [1, 17]. At the excitation powers used in our measurements, we would expect to observe slight bunching near the antibunching dip at a time delay of zero. However, this measurement was limited by noise resulting from low count emitter count rates due to poor mode matching between the waveguide and collection fiber. For completeness, we include in Supplementary Figure 14 the second-order correlation data presented in the main text using a 3-level system fit:

$$g^{(2)}_{3LS}(t) = a\left(1 - (1-b)e^{(-|t-t_{\text{shift}}|)/\tau_1)} + be^{(-|t-t_{\text{shift}}|/\tau_2))}\right), \tag{3}$$

where a and b are fitting parameters, t_{shift} is the temporal location of the antibunching dip, and τ_1 and τ_2 are the antibunching and bunching time constants, respectively.

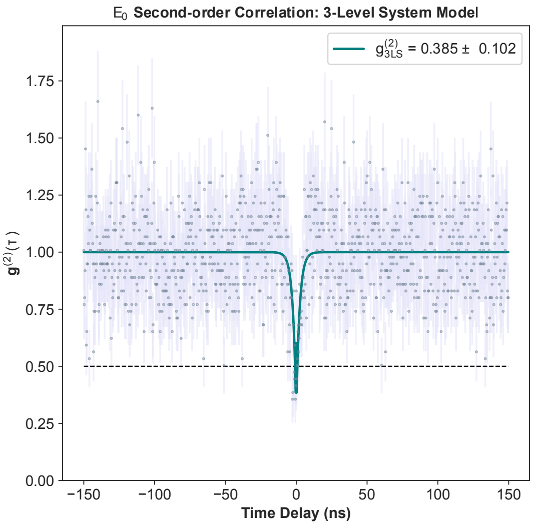

Supplementary Figure 14. **Second-order correlation fit with a three-level model.** The second-order correlation data, measured at 6 K under 10 μW continuous-wave 532 nm excitation power, corresponding to an estimated power density of 5.4 kW$\cdot$cm^{-2}.

[1] W. Redjem, A. Durand, T. Herzig, A. Benali, S. Pezzagna, J. Meijer, A. Y. Kuznetsov, H. S. Nguyen, S. Cueff, J.-M. Gérard, I. Robert-Philip, B. Gil, D. Caliste, P. Pochet, M. Abbarchi, V. Jacques, A. Dréau, and G. Cassabois, Nature Electronics **3**, 738 (2020).

[2] L. A. Coldren, S. W. Corzine, and M. L. Mashanovitch, *Diode Lasers and Photonic Integrated Circuits*, 2nd ed. (Wiley, Hoboken, NJ, USA, 2012).

[3] P. Udvarhelyi, B. Somogyi, G. Thiering, and A. Gali, Physical Review Letters **127**, 196402 (2021).

[4] Y. Baron, A. Durand, T. Herzig, M. Khoury, S. Pezzagna, J. Meijer, I. Robert-Philip, M. Abbarchi, J.-M. Hartmann, S. Reboh, J.-M. Gérard, V. Jacques, G. Cassabois, and A. Dréau, Applied Physics Letters **121**, 084003 (2022).

[5] C. Beaufils, W. Redjem, E. Rousseau, V. Jacques, A. Y. Kuznetsov, C. Raynaud, C. Voisin, A. Benali, T. Herzig, S. Pezzagna, J. Meijer, M. Abbarchi, and G. Cassabois, Physical Review B **97**, 035303 (2018).

[6] M. A. Green, Solar Energy Materials and Solar Cells **92**, 1305 (2008).

[7] J. Degallaix, J. Komma, D. Forest, G. Hofmann, M. Granata, D. Heinert, C. Schwarz, R. Nawrodt, L. Pinard, C. Michel, R. Flaminio, and G. Cagnoli, Classical and Quantum Gravity **31**, 185010 (2014).

[8] M. Asheghi, M. N. Touzelbaev, K. E. Goodson, Y. K. Leung, and S. S. Wong, Journal of Heat Transfer **120**, 30 (1998).

[9] C. J. Glassbrenner and G. A. Slack, Phys. Rev. **134**, A1058 (1964).

[10] L. C. Bassett, F. J. Heremans, C. G. Yale, B. B. Buckley, and D. D. Awschalom, Physical Review Letters **107**, 266403 (2011).

[11] J. Strand, M. Kaviani, D. Gao, A.-M. El-Sayed, V. V. Afanas'ev, and A. L. Shluger, Journal of Physics: Condensed Matter **30**, 233001 (2018).

[12] V. V. Afanas'ev and V. K. Adamchuk, Progress in Surface Science **47**, 301 (1994).

[13] N. Aslam, G. Waldherr, P. Neumann, F. Jelezko, and J. Wrachtrup, New Journal of Physics **15**, 013064 (2013).

[14] D. L. Staebler and C. R. Wronski, Applied Physics Letters **31**, 292 (1977).

[15] M. Stutzmann, W. B. Jackson, and C. C. Tsai, Physical Review B **32**, 23 (1985).

[16] K. M. Lee, K. P. O'Donnell, J. Weber, B. C. Cavenett, and G. D. Watkins, Physical Review Letters **48**, 37 (1982).

[17] M. Hollenbach, Y. Berencén, U. Kentsch, M. Helm, and G. V. Astakhov, Optics Express **28**, 26111 (2020).
